# The effect of interaction between Melanocortin-4 receptor polymorphism and dietary factors on the risk of metabolic syndrome

**DOI:** 10.1186/s12986-016-0092-z

**Published:** 2016-05-14

**Authors:** Gelareh Koochakpoor, Maryam S. Daneshpour, Parvin Mirmiran, Seyed Ahmad Hosseini, Firoozeh Hosseini-Esfahani, Bahareh Sedaghatikhayat, Fereidoun Azizi

**Affiliations:** Department of Nutrition, School of Paramedicine, Ahvaz Jundishapur University of Medical Sciences, Ahvaz, Iran; Cellular Molecular and Endocrine Research Center, Research Institute for Endocrine Sciences, Shahid Beheshti University of Medical Sciences, Tehran, Iran; Nutrition and Endocrine Research Center, Research Institute for Endocrine Sciences, Shahid Beheshti University of Medical Sciences, Tehran, Iran; Faculty of Nutrition Sciences and Food Technology, National Nutrition and Food Technology Research Institute, Shahid Beheshti University of Medical Sciences, Tehran, Iran; Endocrine Research Center, Research Institute for Endocrine Sciences, Shahid Beheshti University of Medical Sciences, Tehran, Iran

**Keywords:** Dietary Factores, MC4R polymorphism, Metabolic syndrome, Interaction

## Abstract

**Background:**

Controversial data is available on the effect of the Melanocortin-4 receptor (MC4R) gene variation on metabolic syndrome (MetS) and ineffectiveness of diet in managing MetS. Effects of the interaction between MC4R polymorphism and dietary factors on MetS were investigated in this study**.**

**Methods:**

Subjects of this nested case-control study were selected from among participants of Tehran Lipid and Glucose Study. Each case (*n* = 815) was pair matched randomly with a control by age (±5 years) and sex from among those who had not developed ≥1 MetS components at the time that the corresponding case developed MetS**.** Dietary patterns were determined using factor analysis on 25 foods groups using a valid and reliable, 168-item semi-quantitative food frequency questionnaire (FFQ). MC4R rs12970134 were genotyped by Tetra-Primer ARMS-polymerase chain reaction analysis. Adjusted conditional logistic regression was used to estimate the interactions of SNP with quartiles of dietary factors in relation to MetS. MetS was defined by the modified National Cholesterol Education Program/Adult Treatment panel III.

**Results:**

Two dietary patterns were extracted. The healthy dietary pattern was loaded heavily on vegetables, legumes, low fat dairy, whole grains, liquid oils and fruits; the western dietary pattern consisted of a high intake of soft drinks, fast foods, sweets, solid oils, red meats, salty snacks, refined grains, high fat dairy, tea and coffee, eggs and poultry. Among A allele carriers, being in the highest quartiles of western dietary pattern score and saturated fatty acid intake had an increased risk of MetS, compared to those in the lowest quartile (P trend = 0.007). Saturated fatty acid intake could modulate the association of A allele carriers of MC4R with MetS (P interaction = 0.03). A significant interaction was observed between rs12970134 with total fat and iron intake on the risk of abdominal obesity (P interaction < 0.05).

**Conclusion:**

Our findings suggest an interaction between rs12970134 and western dietary pattern, fat and vegetable intakes on the risk of MetS or its components.

## Background

Metabolic syndrome (MetS), a combination of phenotypes including abdominal obesity, dyslipidemia, impaired glucose metabolism and high blood pressure [1], is associated with development of diabetes mellitus and cardiovascular disease [2]. Efforts of genetic studies to find the reason for co-occurrence of Mets components led to investigating the likelihood of a role for the A allele of rs12970134, a variant in the melanocortin-4 receptor (MC4R) gene, in the development of MetS [3–5]; however, there are some studies that found no such relationship between variants in the MC4R gene and this syndrome [6, 7]. Several mechanisms may explain this inconsistency, but without doubt, one of these is the role of environmental factors such as diet in modulation of the effects of MC4R gene variation. Moreover, the effectiveness of diet in the treatment of MetS components differs in different ethnicities with variant genetic structures [8]. This reciprocal effect between diet and genetic structure highlights the importance of studying the interaction between MC4R genes with dietary factors. A previous study showed that dietary patterns can modify the effects of APOA1 and APOC3 SNPs genes [9]. The interaction between genotypes of rs17782313 MC4R and nutrient intakes in relation to phenotypes related to obesity has only been examined in a few cross-sectional studies [10]; hence to gain more insight into the etiology of MetS and finding a diet tailored to the genetic makeup of patients with MetS, the effect of interaction between MC4R rs12970134 with dietary patterns, nutrients (macronutrients, Iron, zinc and magnesium) or food groups on the risk of MetS and related components were investigated in this nested case-control study.

## Methods

### Study population

Subjects of this nested case-control study were selected from among participants of the Tehran Lipid and Glucose Study (TLGS), a large-scale, community-based, prospective study being performed on sample of residents of district 13 of Tehran, capital of Iran. The first phase of the TLGS was conducted from 1999 to 2001 in 15,005 subjects, aged ≥3 years, and follow-up examinations have been conducted every 3 years (2002–2005; 2006–2008; 2008–2011 and 2011–2014) to identify newly developed diseases. Details of this ongoing cohort study have been published elsewhere [11, 12].

Of 11,001 and 9807 individuals, aged ≥18 years, who participated in baseline and second follow–up surveys respectively, 5280, were excluded because of having MetS at either baseline or the second follow-up survey. In the current study, from among participants who developed MetS in third (*n* = 918), the fourth (*n* = 827) or fifth (*n* = 1050) phases, 1198 cases were randomly selected. After excluding individuals with a history of cardiovascular events, weight loss or gain >5 kg in the last 6 months, pregnancy and lactating, or those taking any CVD/anticoagulant/steroid or hormonal medication, 1158 cases were included in the study. Each case was individually pair matched randomly with a control by age (±5 years) and sex from among those who had not developed ≥1 MetS components at the time that the corresponding case developed MetS. After excluding cases/controls lacking DNA purification in the range of 1.7 < A260/A280 < 2, and those whose reported energy intake divided by the predicted energy intake did not qualify for the ±3 SD range, finally data of 1630 (815 pairs) with MetS and matched controls remained for analysis.

Informed written consents were obtained from all participants. The study protocol was approved by two ethical committees, the ethical committee of the Research Institute for Endocrine Sciences, Shahid Beheshti University of Medical Sciences, Tehran and the medical research ethics committee of JundiShapour University of Medical Sciences, Ahvaz, Iran.

### Measurements

Dietary intake was assessed with the use of a valid and reliable, 168-item semi-quantitative food frequency questionnaire (FFQ) to assess the usual food intake of individuals during the 12 months before the examination. The consumption frequency of each food item on a daily, weekly or monthly basis was converted to daily intakes; portion sizes were then converted to grams, using household measures. Because the Iranian Food Composition Table (FCT) is incomplete, we used the United States Department of Agriculture (USDA) FCT to analyze foods and beverages [13]; however, the Iranian FCT was used for some national foods and beverages, which are not listed in the USDA FCT [14]. Based on macronutrient composition and using current literature, 25 food groups were categorized [15–17].

Anthropometric assessment was done using a standardized process. Weight was measured to the nearest 100 g, using digital scales while the subjects were minimally clothed and not wearing shoes. Height was measured to the nearest 0.5 cm with a tape measure in a standing position and with shoulders in a normal alignment and without shoes. Waist circumference (WC) was measured to the nearest 0.1 cm, at the umbilical level over light clothing, using an unstretched tape meter without any pressure to body surface. Blood pressure (BP) was measured on the right arm in a sitting position twice, after a 15 min rest in the sitting position and finally, the mean of the two measurements was reported as the participant's BP.

Physical activity level was assessed using the Persian translated modifiable activity questionnaire (MAQ) with high reliability and relatively moderate validity [18]. The frequency and time spent on light, moderate, hard and very hard intensity activities, according to the list of common activities of daily life over the past year were obtained, and the activity data was transformed into metabolic equivalent hours per week (METs/h/wk) [18–20].

Fasting blood samples were taken after 10–12 h of overnight fasting. Fasting plasma glucose (FPG) and triglycerides (TG) were measured by the enzymatic colorimetric method and high density lipoprotein cholesterol (HDL-C) was measured after precipitation of apo-lipoprotein β with phosphotungstic acid. Analyses were performed using Parsazmun kits (Tehran, Iran) and a Selectra 2 autoanalyzer (Vital Scientific, Spankeren, Netherlands).

### Genotyping

Genomic DNA was extracted from peripheral blood using standard salting-out method [21]. The selected polymorphism (rs12970134) was studied by Tetra-primer ARMS method. The primers determined through the national center for biotechnology information (NCBI) site [22]. Our T-ARMS assay with different inner allele specific primers produce allele-specific PCR products; forward: ATA CTG ACT CTT ACC AAA CA AAG CAC GAA and reverse: AGC ACC CTT CTG ATA AAT CTT TGT TAG C. Two outer primers produce a PCR product to be used as an internal control for reaction; forward: AGT AAG AGT GAA GAT TTG AGG GAT GGA GA and reverse: TCT CTT CGA GGA GTG TTT GAG TCT GA. For the SNP mentioned, the PCR reaction was optimized in a 12.5 μl total volume containing 1.5 μl DNA template, 6.25 μl master mix containing MgCl2, Smart Taq polymerase (CinnaGene co; Iran), BSA 0.1 % (TaKaRa; Japan), 2 μl primer containing (outers and inners) and 2.75 μl Water.

The PCR amplification was carried out with an initial denaturation at 94 °C for 3 min, denaturation at 95 °C for 30 s (35 cycles), 45 s of annealing at 63.13 °C (35 cycles), 1 min of extension at 72 °C and an additional 10 min of extension at 72 °C at the end of the final cycle. PCR products were resolved by electrophoresis in a 1.8 % agarose gel, this procedure which rendered three bands in heterozygotes (614, 372 and 298 bp) and two bands in homozygotes (mutant allele carrier resulting in 614 and 298 bp, wild carrier allele resulting in 614 and 372 bp). A 614 bp band was always obtained as the control for the success of the amplification. To validate the accuracy of genotype scoring by tetra-primer ARMS-PCR, the three (614, 372 and 298 bp) fragments were directly sequenced.

### Definitions

MetS was defined according to the modified definition of the National Cholesterol Education Program/Adult Treatment panel III (ATP III) [23], as having three or more of the following criteria: 1) abdominal obesity, WC ≥ 95 cm for both genders, according to the newly-introduced cut-off points for Iranian adults [24], 2) high TG (≥150 mg/dL) or drug treatment, 3) low HDL-C (<40 mg/dL in men or < 50 mg/dL in women) or drug treatment, 4) high blood pressure (SBP/DBP ≥ 130/85 mmHg) or antihypertensive drug treatment and 5) high FPG (≥110 mg/dL) or drug treatment for elevated glucose.

### Statistical analysis

For the descriptive analysis, a comparison of qualitative and quantitative variables between cases and controls was done, using the student t and Chi square test statistics respectively; TG concentration (a non-symmetric quantitative variable) was log-transformed before the statistical analysis. The genotype and allele frequencies for the analyzed polymorphism were obtained using Power-Marker software. Pearson’s Chi-square statistic was used to calculate the Hardy-Weinberg equilibrium.

Dietary patterns were identified using factor analysis with varimax rotation, based on 25 food groups. Dietary patterns were extracted based on the eigenvalues (>1), scree plot, factor interpretability, and the variance explained (>5 %).

Conditional logistic regression was used to estimate the interactions of SNP with quartiles of dietary factors in relation to MetS after adjustment for baseline BMI. Two likelihood scores were obtained performing this statistical analysis, with and without the interaction terms; *P* value for interaction was determined by performing the likelihood ratio test.

Conditional logistic regression was used to generate odds ratios (ORs) for MetS for individuals with the carrier or non carrier of risk allele (GG/AG + AA) across quartiles of dietary pattern scores, food groups and nutrients intake (Q1-Q4). The lowest quartile of dietary factors and the homozygote group with major allele were examined as the reference group. Unconditional logistic regression was performed to estimate the interactions of MC4R SNP with quartiles of dietary pattern scores, food groups and nutrients intake in relation to MetS components. All ORs were adjusted for variables proven to be associated with MetS components, including age, gender, educational level, smoking status, physical activity and energy intake. To determine the *P* value for trend across the quartiles of dietary factors, logistic regression was used, using the median of each quartile of dietary pattern scores as a continuous variable. Data were analyzed using STATA statistical package v.12.0 or SPSS Version 16.0 (Version 16.0; SPSS, Chicago, IL).

## Results

### Study population

General characteristics of participants by cases and controls are shown in Table 1. There was no significant difference between the two groups in physical activity, years of education, smoking or daily energy intake. However, the values of MetS components were significantly different among case and controls at the beginning of the study. Genotype frequency was in Hardy Weinberg equilibrium (*P* = 0.92). No significant differences were observed in the frequencies of genotypes or alleles between the two groups.

**Table 1:** Characteristics of the study population in subjects with metabolic syndrome (MetS) cases and controls

	Without MetS (*n* = 815)		With MetS (*n* = 815)	
		SD		SD
Baseline Age (y)†	43.03	12	43.31	11
Men	44.4	12	41.8	12
Women	43.7	11	42.9	11
Current smokers (%)	21.7		20.7	
Physical activity (MET.h/w)	7.46	12	7.34	13
Education level ≥14 years (%)	11.9		9.5	
Baseline BMI^a^ (Kg/m^2^)	24.0	4	28.1*	4
Obesity (%)^b^	16.0		47.2*	
Baseline WC (cm)	83.2	10	93.2*	11
Abdominal obesity (%)^c^	54.0		90.7*	
Baseline systolic BP (mmHg)	112.4	15	121.88*	17
Baseline diastolic BP (mmHg)	73.8	8	79.6*	8
Elevated BP (%)^d^	20.6		58.3*	
Baseline HDL-C (mg/dl)	58.9	9	44.8 *	10
Low HDL-C (%)^e^	28.7		82.6*	
Baseline TG (mg/dl)	104.5	42	174.1*	71
High TG (%)^f^	14.0		68.0*	
Baseline FBG (mg/dl)	87.0	12	109.95*	10
High FBG (%)^g^	21.4		79.1*	
Energy intake (Kcal/day)	2414	1072	2410	878
Carbohydrate (% of energy)	59.1	8	59.4	9
Total fat (% of energy)	30.1	8	29.9	7.2
Saturated fat (% of energy)	10.1	3	9.8	3
MUFA (% of energy)	10.0	3	10.0	2
PUFA (% of energy)	6.0	2	6.1	2
Allele frequency rs12970134 n (%)				
G	1038 (63)		989 (60)	
A	592 (36)		641 (39)	
Genotype frequency rs12970134 n (%)				
GG	330 (40.5)		297 (36.4)	
AG	378 (46.6)		395 (48.5)	
AA	107 (13.1)		123 (15.1)	

*BMI* body mass index, *WC* waist circumference, *BP* blood pressure, *HDL-C* high density lipoprotein cholesterol, *TG* triacylglycerol, *FBG* fasting blood glucose, *MUFA* mono-unsaturated fatty acids, *PUFA* poly-unsaturated fatty acids

*P < 0.05; †values are mean unless otherwise listed. ^a^baseline means at first phase or second phase of TLGS. ^b^BMI ≥30 kg/m2, ^c^WC ≥ 95 cm for both genders. ^d^BP ≥130/85 mmHg, ^e^HDL-C <40 mg/dl in men and <50 mg/d in women; ^f^TG ≥ 150 mg/dl, ^g^FBG ≥110 mg/dl

Two major dietary patterns were identified; the healthy dietary pattern was loaded heavily on vegetables, legumes, low fat dairy, whole grains, fruit juices, liquid oils and fruits, whereas the western dietary pattern consisted of high intakes of soft drinks, fast foods, sweets and sugar, solid oils, red meats, salty snacks, refined grains, high fat dairy, tea and coffee, eggs and poultry (Table 2).

**Table 2:** Factor loadings for the two dietary patterns identified in study participants

Food groups	Dietary patterns^a,b^	
	Western	Healthy
Soft drinks	0.573	
Sweets and Sugar	0.559	
Fast foods	0.506	
Solid oils	0.455	
Red meats	0.451	
Salty snacks	0.447	
Refined grains	0.443	
High fat dairy	0.393	
Eggs	0.369	
Tea and coffee	0.326	
Poultry	0.251	
Non Starchy vegetables		0.650
Starchy vegetables		0.610
Legumes		0.589
Low fat dairy		0.466
Whole grains		0.436
Fruit juice	0.201	0.412
Liquid oils	0.315	0.342
Fruits		0.262
Fish		
Nuts and seeds		
Variance (%)	11.186	10.366

^a^Values are factor loadings of dietary patterns measured by factor analysis. Factor loading below ±0.2 are not shown in the table for simplicity. ^b^Eigenvalues > 1, KMO: 0.75

### Interactions of SNP and dietary patterns in relation to MetS or its components

After adjustment for baseline BMI, there was a significant interaction between MC4R SNP and the western dietary pattern in relation to MetS (P interaction = 0.04). The risk of MetS was not homogenous in the two rs12970134 MC4R genotype groups, across quartiles of western dietary pattern scores. Among A allele carriers (AG + AA), being in the highest quartiles of western dietary pattern score had an increased risk of MetS, compared with those in the lowest quartiles (P trend = 0.007); however in the GG genotype carriers of rs12970134, no significant association was observed between the western dietary pattern score and risk of MetS (P trend = 0.32) (Table 3). There was no significant interaction between MC4R SNP and healthy dietary pattern in relation to MetS. Also, no interaction was observed between MC4R gene and dietary patterns in relation to MetS components.

**Table 3:** Adjusted ORs (95 % CI) for metabolic syndrome according to quartiles of dietary pattern scores^a^

	Q1	Q2	Q3	Q4	P for trend	P for interaction
Western dietary pattern						0.04
GG	1.	1.37 (0.77–2.45)	1.21 (1.09–2.11)	1.41 (0.77–2.17)	0.32	
GA + AA	0.75 (0.41–1.35)	1.25 (0.75–2.08)	1.49 (0.87–2.53)	1.71 (1.04–2.41)	0.007	
Healthy dietary pattern						0.82
GG	1	1.16 (0.68–1.37)	1.20 (0.67–2.13)	0.89 (0.51–1.53)	0.93	
GA + AA	0.87 (0.49–1.54)	1.15 (0.68–1.94)	1.21 (0.74–2.08)	1.29 (0.77–2.17)	0.41	
SFA						0.03
GG	1	1.77 (1.05–2.34)	1.10 (0.67–1.83)	0.83 (0.47–1.47)	0.50	
GA + AA	0.90 (0.50–1.64)	1.24 (0.74–2.08)	1.43 (0.87–2.36)	1.76 (0.99–2.14)	0.01	

*OR* odds ratio, *Q* quartiles of dietary pattern scores or SFA (Q1 < 7.75, Q2:7.75–9.52, Q3:9.52–11.51 and Q4 > 11.51 % of energy), *SFA* saturated fatty acid

^a^ORs (95 % CI) were calculated by using conditional logistic regression model, adjusted for baseline BMI and the interaction term (SNP × dietary pattern scores)

Participants were matched classified (8 groups) according to quartiles of dietary pattern scores and rs12970134 genotypes

The lowest quartile of dietary pattern scores and homozygote genotype of major allele were used as the reference group

### Interactions of SNP with nutrients and food group intakes in relation to MetS or its component

**Fig. 1:**
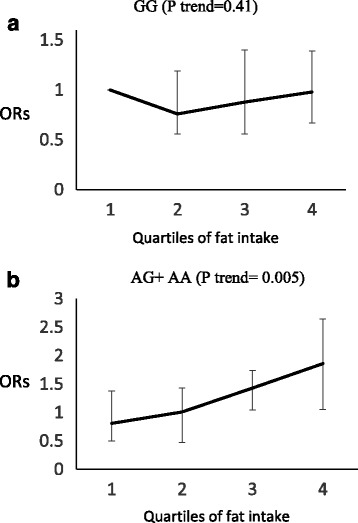
Adjusted OR for abdominal obesity to quartiles of fat intake by MC4R genotypes (p interaction = 0.01). Q1 < 25.23 %, Q2 = 25.23–29.30 %, Q3 = 29.30–35.83 %, Q4 > 35.83 % of energy. In addition, there was a significant interaction between total fat intake and MC4R SNPs in relation to abdominal obesity (P interaction = 0.005), such that in A allele carriers, the risk of abdominal obesity increased across quartiles of total fat intake (P trend = 0.005), an association however not significant in GG homozygote carriers (P trend =0.41)

Among macronutrients examined in this study [carbohydrate, protein, total fat, saturated fatty acid (SFA), Polyunsaturated fatty acids and monounsaturated fatty acid], only SFA intake could modulate the association of risk genotype carriers of MC4R with MetS (P interaction = 0.03). Based on our observations, A allele Carriers (AG + AA) are more sensitive to SFA than carriers of the GG genotypes and A allele carriers, who consumed more SFA had a higher risk of developing MetS than carriers of the GG genotypes, of whom no such relationship was observed. In addition, there was a significant interaction between total fat intake and MC4R SNPs in relation to abdominal obesity (P interaction = 0.01), i.e., in A allele carriers, the risk of abdominal obesity increased across quartiles of total fat intake (P trend = 0.005), however the association was not significant in GG homozygote carriers (P trend = 0.41) (Fig. 1). The significant interaction between other macronutrients and MC4R SNP in relation to other components of MetS was not observed.

**Fig. 2:**
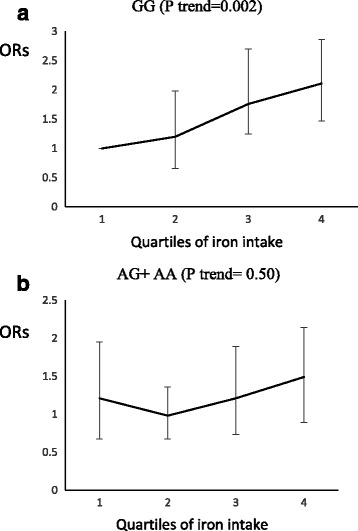
Adjusted OR for abdominal obesity to quartile of iron intake by MC4R genotypes (p interaction = 0.002). Q1 < 13.88 mg, Q2 = 13.88–15.98 mg, Q3 = 15.98–40.36 mg, Q4 > 40.36 mg. Among other nutrients (iron, zinc and magnesium), iron has an interaction with this SNP in the development of abdominal obesity (P interaction = 0.006); in A allele carriers, the risk of abdominal obesity did not change significantly with increased iron consumption (P trend = 0.50) but for GG genotype carriers, more consumption of iron led to an increase in risk of abdominal obesity (P trend = 0.002)

Among nutrients (iron, zinc and magnesium), Iron had an interaction with MC4R SNP in the development of abdominal obesity (P interaction = 0.006); in A allele carriers, the risk of abdominal obesity did not change significantly with increased iron consumption (P trend = 0.50) whereas for GG genotype carriers, higher consumption of iron led to an increase in the risk of abdominal obesity (P trend = 0.002) (Fig. 2). No significant interaction was observed for MC4R SNP with magnesium and zinc intakes, in relation to MetS or its components.

**Fig. 3:**
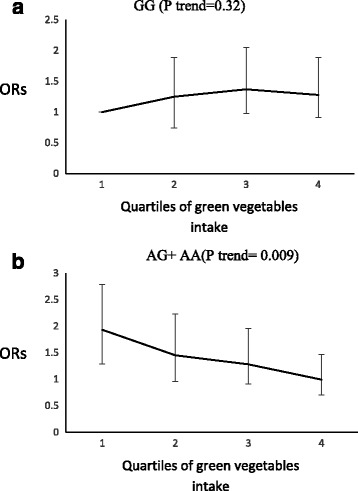
Adjusted OR for low high density lipoprotein cholesterol to quartiles of green vegetable intakes by MC4R genotypes, (p interaction = 0.04). Q1 < 28.43, Q2 = 28.43–45.06, Q3 = 45.06–74.08, Q4 > 74.08 gr. The odds ratio for low HDL decreased significantly with increased consumption of green vegetables by the risk genotype carriers (p trend = 0.009), but consumption of this food group had no significant effect on the odds ratio for low HDL in GG genotype carriers (p trend = 0.32)

**Fig. 4:**
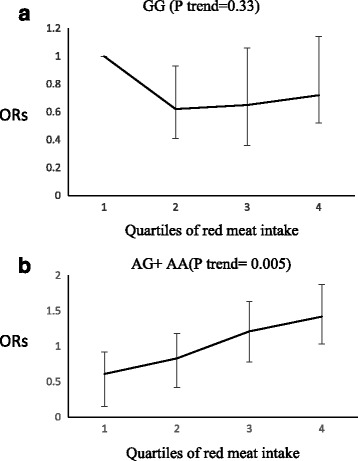
Adjusted OR for high triglycerides to quartiles of red meat intake by MC4R genotypes (P interaction = 0.05); Q1 < 8.02, Q2 = 8.02–13.99, Q3 = 13.99–27.81, Q4 > 27.81 gr. In A allele carriers, more consumption of red meat led to a higher risk of high TG (p trend = 0.005), but this effect was not observed for GG genotype carriers (p trend = 0.33)

**Fig. 5:**
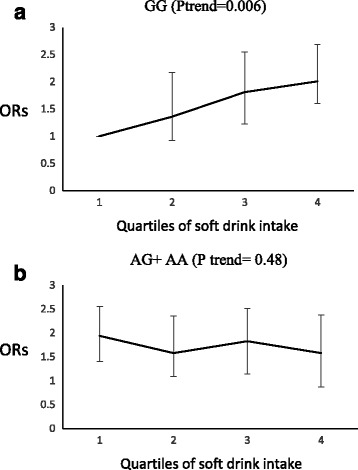
Adjusted OR for high fasting blood glucose to quartiles of soft drink intake by MC4R genotypes (P interaction = 0.02); Q1 < 1.09, Q2 = 1.09–8.33, Q3 = 8.33–28.57, Q4 > 28.57 gr. In GG genotype carriers, the odds ratio for high FPG increased significantly across quartiles of soft drink intakes (p trend = 0.006), but this association was not significant in risk genotype carriers (AG + AA) (p trend = 0.48)

A significant interaction was observed between rs12970134 and green vegetables, soft drink and red meat groups on the risk of low HDL-C (P interaction = 0.04), high FPG (*P* interaction = 0.02) and high TG (P interaction = 0.05), respectively. The odds ratio for low HDL-C decreased significantly with increased consumption of green vegetables by the risk genotype carriers (P trend = 0.009), whereas this food group had no significant effect on the odds ratio for low HDL-C in GG genotype carriers (P trend = 0.32) (Fig. 3). In A allele carriers, increased consumption of red meat led to a higher risk of high TG (P trend = 0.005), an effect not seen for GG genotype carriers (P trend = 0.33) (Fig. 4); in GG genotype carriers, the odds ratio for high FPG increased significantly across quartiles of soft drink intakes (P trend = 0.006), although this association was not significant in AG + AA risk genotype carriers (P trend = 0.48) (Fig. 5).

## Discussion and conclusions

The present study provides information on the effect of the interaction between dietary patterns, nutrient or food groups and genetic polymorphism of MC4R (rs12970134) in association with MetS and its components. Western dietary pattern and SFA consumption could alter the association between the MC4R rs12970134 polymorphism and MetS. Participants with the AG + AA genotype exhibited a higher MetS risk with increasing quartiles of the western dietary pattern score and higher consumption of SFAs but there was no significant interaction between MC4R SNP and the healthy dietary pattern in relation to MetS. Interaction between MC4R genes and dietary patterns has also been reported by Azorin et al., who found that adherence to the Mediterranean diet (as a model of healthy pattern) was effective in reducing the risk of type 2 diabetes risk in carriers of the variant alleles for MC4R rs17782313 [25]; thus the moderating effect of the Mediterranean diet can be attributed to its components, viz olive oil, nuts and fish, compounds that have low factor loadings in these healthy dietary patterns. Our results on western dietary pattern and MetS are consistent with those of another study, in which intake of western dietary pattern were associated with diabetes risk among men with a higher genotype risk score [26]. Increase in fat intake due to fast food, red meat and high fat dairy consumption may be the major agent responsible for the interactions between a western dietary pattern and genetic variation.

Our findings indicate that individuals with susceptible genotypes were more sensitive to total fat intake than individuals with GG genotypes and increased consumption of fat or high fat foods (e.g., red meat) in this genotype group further increased the risk of MetS components.

Several mechanisms could justify the role of fat intake in exacerbating the effect of high risk genotypes on MetS components. Since MC4R functions affected by anorexigenic hormones (insulin and leptin), exposure to high-fat diet with increasing inflammation in the hypothalamus and subsequently increase the resistance to leptin and insulin, leading to reduced MC4R function [27, 28]. Animal studies also suggest that a high-fat diet can influence epigenetic phenomena by changing DNA methylation status of the MC4R gene, and in this way it can change MC4R gene expression level [29]; last but not least, in several studies expression and function of MC4R gene was affected by dietary fat intake, showing that exposure to high-fat diet in rats leads to decreased MC4R mRNA [30, 31].

Previous data report a direct relationship between sugar-sweetened beverage consumption, iron intake and the risk of MetS components [32, 33]. In our study however this relationship was observed in subjects with the GG genotype but not in carriers of the A allele. Since iron deficiency is a frequent finding in obesity [34], the hypothesis arises whether iron metabolism may be altered in carriers of A allele. To confirm this hypothesis, and to clarify how this change in metabolism occurs, further studies are definitely needed.

In the current study, green vegetable consumption modified the effect of genetic variation in HDL-C levels. Change in genetic expression or methylation status of DNA by folate intake or phytochemicals could be a justification for this effect, since no significant interaction was observed between magnesium intake and MC4R rs12970134 in relation to MetS and its components in our study.

The strengths of our study include the use of prospective studies with long-term follow-up, large numbers of cases, matched individually by age and sex, extensive adjustment for potential confounders, use of a dietary pattern analysis to better detect the association of the overall diet composition and finally, selection new cases of MetS, which reduced the possibility of any dietary behavior changes due to awareness of disease.

However, several limitations need to be addressed; our study population was highly homogeneous, as the study was performed only on residents of district 13 of Tehran. Statistical methods used to define dietary patterns, i.e., factor analysis, are somewhat subjective and dietary patterns can vary by socioeconomic status, ethnic groups, and cultures. However, previous studies have shown reasonable reproducibility, validity and stability of the western dietary pattern in a subsample of a Tehranian population [35]. Adiponectin measurement and genotyping of the FTO polymorphisms were not conducted in this study, although adipocytokines such as adiponectin and the FTO gene are effective in Mets. Insulin sensitivity, as a sensitive marker, was not measured, which is why we were unable to detect interaction of dietary factors and MC4R SNP in relation to it.

The present study demonstrated a significant interaction between the western dietary pattern and dietary SFA with MC4R variant in relation to MetS; high intake of SFA (about 14 % of energy) by A allele carriers increased the risk of MetS, however consuming the same amount of SFA by GG genotype carriers had no effect on the risk of Mets. Also a significant interaction was observed between MC4R SNPs and green vegetables, soft drink, red meat and iron intakes respectively on the risk of low HDL-C, high FPG, and high TG and abdominal obesity, so early detection of people with the high-risk MC4R rs12970134 allele and limiting the western dietary pattern and fat intake and eating more vegetables would be a suitable strategy to reduce the incidence of MetS or its components in this genetic group.

### Ethics approval and consent to participate

The study protocol was approved by two ethical committees, the ethical committee of the Research Institute for Endocrine Sciences, Shahid Beheshti University of Medical Sciences, Tehran and the medical research ethics committee of JundiShapour University of Medical Sciences, Ahvaz, Iran.

### Consent for publication

Not applicable.

## Abbreviations

CVD: Cardiovascular Disease
FCT: Food Composition Table
FFQ: Food Frequency Questionnaire
FPG: Fasting Plasma Glucose
MAQ: Modifiable Activity Questionnaire
MC4R: Melanocortin-4 receptor
MetS: Metabolic Syndrome
ORs: Odds Ratio
TG: Triglycerides
TLGS: Tehran Lipid and Glucose Study
WC: Waist Circumference
